# Correlation of cadherin-17 protein expression with clinicopathological features and prognosis of patients with sporadic gastric cancer

**DOI:** 10.1590/1414-431X20154645

**Published:** 2015-09-29

**Authors:** W. Meng, T. Gu, L. M. Gao, Z. G. Zong, L. Meng, Z. Z. Fu, L. Guo

**Affiliations:** 1 Department of Medical Oncology, The First Affiliated Hospital, Hebei North University, Zhangjiakou, Hebei Province, China; 2 Department of Medical Oncology, The First Hospital of Qinhuangdao, Qinhuangdao, Hebei Province, China; 3 Department of Orthopaedics, The First Affiliated Hospital, Hebei North University, Zhangjiakou, Hebei Province, China; 4 Department of Anesthesiology, Guilin Medical University, Guilin, Guangxi Province, China; 5 Department of Orthopedics, Jinling Hospital, School of Medicine, Nanjing University, Nanjing, Jiangsu Province, China

**Keywords:** Cadherin-17, Gastric cancer, Clinicopathological features, Prognosis, Meta-analysis, Cohort study

## Abstract

This study aimed to explore the correlations between cadherin-17 (CDH17) protein expression and the clinicopathological features and prognosis of patients with sporadic gastric cancer (GC). Nine relevant studies of 1,960 patients were identified using electronic database searches supplemented with a manual search in strict accordance with inclusion and exclusion criteria. Statistical analyses were conducted using STATA 12.0 statistical software. Relative risks and 95% confidence intervals were determined, and *Z* test was used to measure the significance of the overall effect size. A total of nine eligible cohort studies were included in this meta-analysis. The expression of CDH17 in patients with diffuse GC was significantly higher than in those with intestinal-type GC. Moreover, the tumor depth of invasion differed significantly between patients with positive CDH17 (CDH17+) and negative CDH17 (CDH17-) GC. However, there were no significant differences between CDH17+ and CDH17- GC patients with respect to tumor node metastasis clinical stages, histological grades, or lymph node metastasis. Despite the differences in invasive depth, there was no significant difference in 5-year survival rates between CDH17+ and CDH17- GC patients. Our meta-analysis provides evidence that CDH17 protein expression may be associated with the development of GC, suggesting that CDH17 is an important biomarker that could be useful for the early diagnosis of GC. However, CDH17 levels do not appear to impact overall survival.

## Introduction

Gastric cancer (GC) is the fourth most common cancer and the second main cause of cancer-related deaths worldwide (1,2). Compared with economically developed countries, its incidence and mortality is higher in developing countries, with the highest incidence rates reported in Eastern Europe, Eastern Asia, and South America (3). Epidemiological evidence shows that GC has become the fourth most common cancer in men (after lung, prostate, and colorectal cancers) and the fifth in women (after breast, colorectal, cervical, and lung cancers). Approximately 464,000 men and 273,000 women were estimated to have died from GC in 2011 (4,5). GC patients often have poor outcomes that account for 10% of total cancer deaths, and radical surgeries because of limited treatment options, while more than 50% of GC patients recur (6).

The most important clinicopathologic prognostic factors for GC are tumor location, depth of tumor invasion, and lymph node involvement (7). Prospective studies also demonstrated that the interaction between genetic and environmental factors may be involved in the etiology of GC (8,9). Environmental factors include nutritional factors such as obesity, high salt consumption, low intake of fresh fruits and vegetables, high caloric consumption, and high nitrate consumption, occupational factors such as exposure to rubber and coal, and other factors such as cigarette smoking and alcohol consumption (2). In recent years, several intrinsic genetic factors such as expression of the cadherin-17 gene (*CDH17*) have been implicated in the carcinogenesis and progression of human cancers, and have become a popular research topic (10). Indeed, some studies suggested that CDH17 participates in tumor invasion and metastasis and may be a valuable marker for the diagnosis and evaluation of GC (11,12).

Cadherins, one of the adhesion molecule families, play a leading role in mediating cell-cell adhesion, and are important in tumorigenesis (13). CDH-17, also known as human peptide transporter-1 or liver-intestine cadherin, is regarded as a structurally unique member of the cadherin superfamily and regulates intercellular adhesion because it can retain its adhesive function without interacting with other cytoplasmic components (7,14). The biological function of CDH17 remains unknown, although many studies have demonstrated elevated CDH17 levels in various human cancers, and linked it to prognosis and risk evaluation (15). CDH17 has also been reported to be expressed in human intestinal and pancreatic ductal epithelial cells, while the overexpression of CDH17 was detected in colorectal cancer, hepatocellular carcinoma, and pancreatic cancer (14). Serial studies have reported that the overexpression of CDH17 in GC is associated with tumor node metastasis (TNM) and deeper invasion, and it could be regarded as an independent prognostic marker in undifferentiated and stage II or III GC (11,12,16). However, other reports indicate that it participates in the development of GC so it may be a promising prognostic marker for early-stage GC (15). In light of these conflicting suggestions, we performed a meta-analysis of all available data to assess the potential role of CDH17 protein expression in the development and prognosis of GC.

## Material and Methods

### Literature search

To identify all studies that assessed the correlations between CDH17 protein expression and the clinicopathological features and prognosis of patients with GC, we comprehensively searched databases Ovid, PubMed, EBSCO, SpringerLink, Wiley, Web of Science, China National Knowledge Infrastructure (CNKI) database, Wanfang database, and VIP database (last updated search in October 20, 2014) using the following common selected keywords: (“stomach neoplasms” or “gastric cancer” or “stomach cancer” or “gastric neoplasms” or “gastric carcinomas” or “stomach carcinomas” or “stomach neoplasms”) and (“CDH17 protein, human” or “CDH17” or “liver-intestine-cadherin” or “cadherin-17”). We also manually reviewed potential relevant articles identified using related search engines.

### Inclusion and exclusion criteria

After carefully reading the abstracts and full articles, studies were included if they met the following inclusion criteria: 1) the study was a non-randomized clinical cohort trial investigating the correlation between CDH17 protein expression and the clinicopathological features and/or prognosis of GC; 2) the study enrolled patients diagnosed with sporadic GC, which was confirmed by histopathologic examinations using tissue samples for CDH17 detection collected from the edge of the tumor; 3) the article contained sufficient information about CDH17 expression levels; 4) the study used immunohistochemistry to quantify CDH17 expression; 5) the final outcome of the study included a clinical stage, histological grade, and 5-year survival rate, and 6) the study was either in Chinese or English. If studies were identified that were written by the same author, only the latest or most complete study was included. Exclusion criteria were: 1) the literature data lacked integrity; 2) the article was an abstract, review, case report, letter, meta-analysis, or proceedings; and 3) the study was a repeated publication or a study with data that overlapped with another study.

### Data extraction

With the aim of reducing bias and increasing credibility, two investigators independently collected information from the enrolled papers based on the selection criteria and reached a consensus on all the items through discussion. The following relevant data were collected from eligible studies, although several articles did not contain all of the data: surname of first author, time of publication, country and ethnicity of subjects, language, age and gender of subjects, study design, number of samples, 5-year survival rate, Lauren grade, TNM stage, histologic grade, invasive grade, and lymph node metastasis (LNM).

### Quality assessment

To determine whether the methodological quality of the study in question was high, the two authors used a set of predefined criteria based on those of the Critical Appraisal Skills Programme (CASP; http://www.casp-uk.net/). The CASP criteria are scored based on these aspects: if the study determines a clearly focused issue (CASP01); if the cohort studies are recruited in an acceptable way (CASP02); if the exposure is accurately measured to reduce bias (CASP03); if the outcome is precisely measured to reduce bias (CASP04); a) if the authors take all important confounding factors into consideration; b) if they take account of the confounding factors in the study design (CASP05); a) if the follow-up of the subjects is adequate; b) if the follow-up of the subjects is sufficiently long (CASP06); the results of the study (CASP07); if the results are precise (CASP08); if the results are reliable (CASP09); if the results can be applied to the local population (CASP10); if the results are consistent with other evidence (CASP11); and the implications of this trial for practice (CASP12). Discrepancies on CASP scores of the included articles were resolved by discussion and consultation with a third reviewer.

### Statistical analysis

Statistical analyses were carried out using the STATA statistical software (version 12.0, Stata Corporation, USA). To assess the correlations between CDH17 protein expression and the clinicopathological features and prognosis of GC patients, relative risk (RR) and 95% confidence intervals (95%CI) were calculated using a random effects or fixed effects model. The statistical significance of pooled RRs was estimated using a *Z*-test. We used Cochran’s Q-test (P<0.05 was considered significant) and the *I* ^2^ test to assess heterogeneity among the studies (17). A random effects model was applied when there was evidence of significant heterogeneity (P<0.05 or *I* ^2^ test >50%). Otherwise, a fixed effects model was used (18,19). We also applied a sensitivity analysis to evaluate if a single study had adequate weight to impact on the overall estimate. Further, the effect of publication bias was detected by Egger’s linear regression test (P<0.05 was considered significant), which can be used to evaluate funnel plot asymmetry, suggesting a possible publication bias (20,21). Univariate and multivariate meta-regression analyses were applied to assess the potential sources of heterogeneity. Further identification was conducted using a Monte Carlo method (22).

## Results

### Baseline characteristics of all included studies

**Figure 1:**
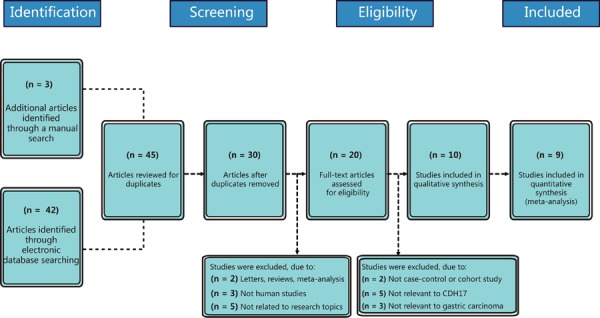
Flow chart showing the detailed study inclusion and exclusion procedures. Nine cohort studies were included in this meta-analysis.

**Figure 2:**
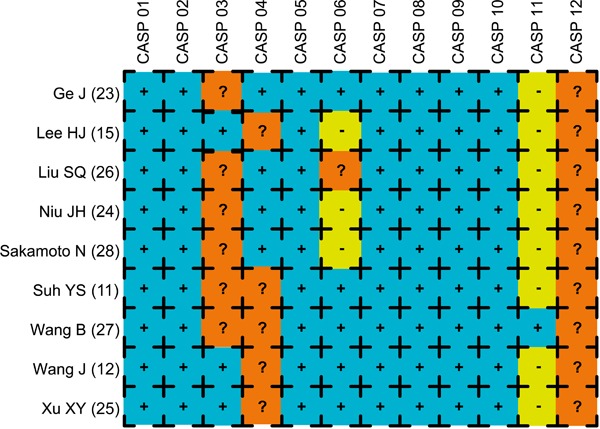
The Critical Appraisal Skills Programme (CASP) score for assessing the methodological quality of the nine enrolled cohort studies. CASP: <http://www.casp-uk.net/>.

A total of 45 published studies were identified through electronic and manual database searches. Figure 1 illustrates the processes of literature screening and selection. After excluding duplicates (n=15), letters, meta-analyses, reviews (n=2), non-human studies (n=3), and studies irrelevant to the research topic (n=5), the remaining 20 studies were examined. Subsequently, 10 further studies were excluded because they were not cohort studies (n=2), did not correlate CDH17 with desired measures (n=5), or were not associated with GC (n=3). After the remaining 10 articles were further reviewed, one was excluded for incomplete data so a total of nine studies including 1,960 patients with GC were included in this meta-analysis. All included articles were published between 2008 and 2012 (11,12,15,23-28). Seven of the studies included Asian subjects and two trials included Caucasian subjects. Six trials were from China, one was from Japan, and two were from the United States. Sample sizes ranged from 46 to 440. The CASP quality score and baseline characteristics of included studies are shown in Figure 2and Table 1, respectively.

**Table:**
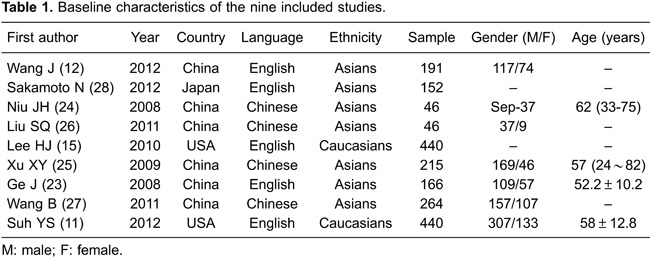

### Correlation between CDH17 and pathological characteristics of GC

**Figure 3:**
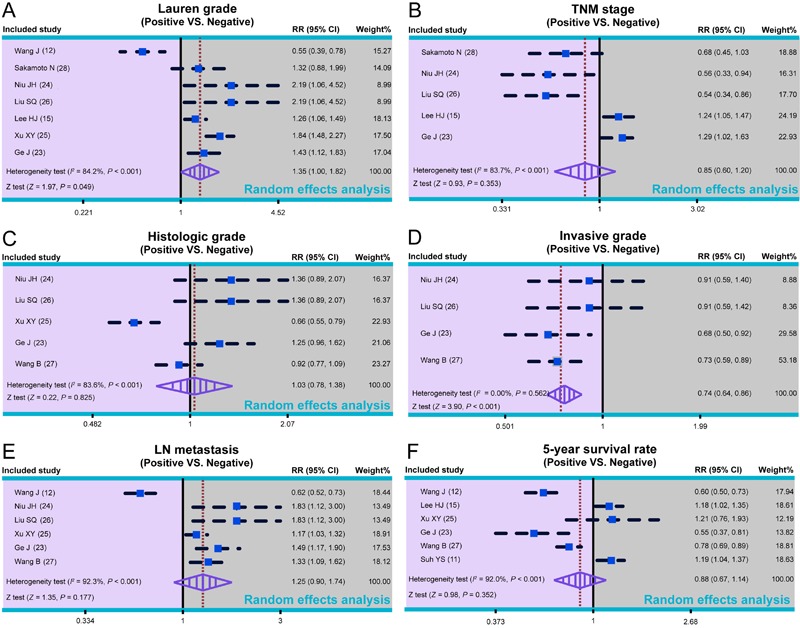
Forest plots of the correlation between cadherin-17 (CDH17) protein expression and the prognosis of patients with gastric cancer (GC).

All studies included in this meta-analysis reported an association between CDH17 and GC. The heterogeneity test revealed that heterogeneity existed across studies with respect to Lauren classification, TNM stage, histological grade, and LNM of GC (Lauren classification: I^2^=84.2%, P<0.001; TNM stage: I^2^=83.7%, P<0.001; histological grade: I^2^=83.6%, P<0.001; LNM: I^2^=92.3%, P<0.001). Therefore, a random effects model was used. There was no heterogeneity among the four studies associated with invasive depth, so a fixed effects model was used in these cases. Our meta-analysis indicated that, based on the Lauren classification, positive expression of CDH17 in patients with diffuse GC was significantly higher than in intestinal-type GC (RR=1.35, 95% CI=1.00-1.82, P=0.049). There was also a significant difference in the depth of invasion between GC patients with positive CDH17 expression (CDH17+) and those with negative CDH17 expression (CDH17-) (RR=0.74, 95% CI=0.64-0.86, P<0.001). However, there was no significant difference between CDH17+ and CDH17- GC patients with respect to TNM clinical stage, histological grade, or LNM (all P>0.05; Figure 3).

### Correlation between CDH17 and prognosis of GC patients

A total of six studies reported an association between CDH17 expression and the prognosis of GC patients. Because heterogeneity was observed among the studies related to the 5-year survival rate of GC patients (I^2^=92.0%, P<0.001), a random effects model was used. This meta-analysis indicated that there was no significant difference in the 5-year survival rates between CDH17+ and CDH17- GC patients (RR=0.88, 95%CI=0.67-1.14, P>0.05), as shown in Figure 3.

### Sensitivity analysis and publication bias

**Figure 4:**
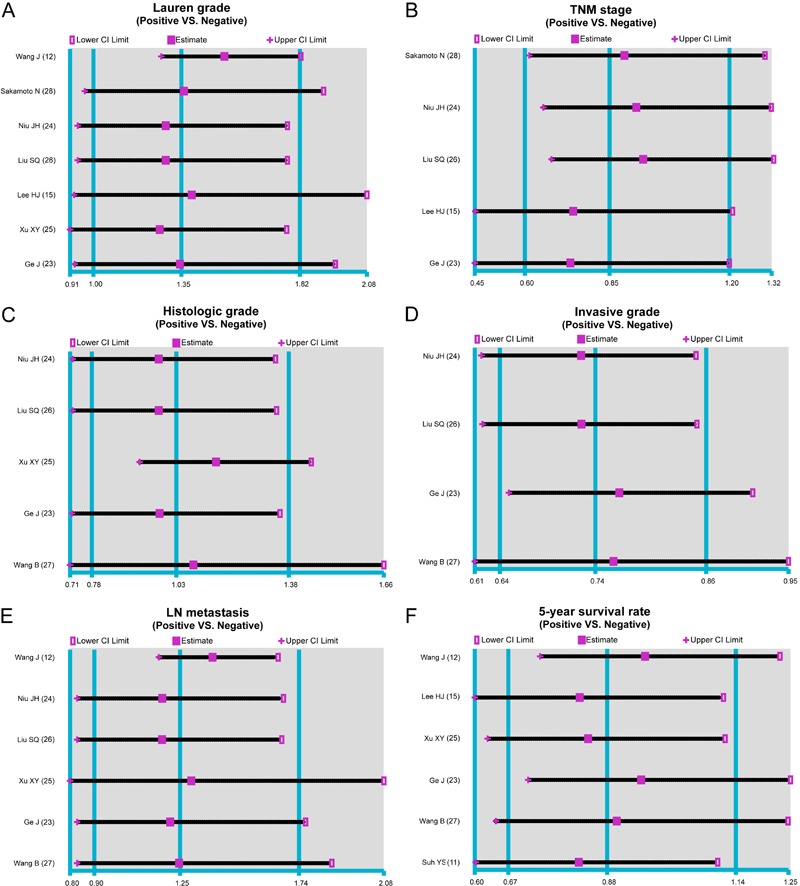
Sensitivity analyses of the correlation between cadherin-17 (CDH17) protein expression and the prognosis of patients with gastric cancer (GC).

**Figure 5:**
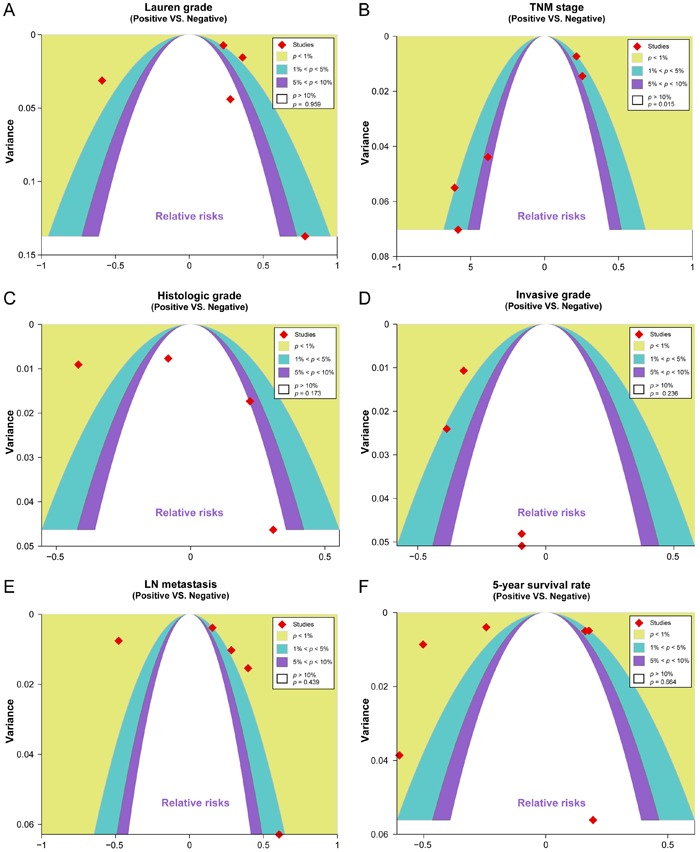
Publication bias of the correlation between cadherin-17 (CDH17) protein expression and the prognosis of patients with gastric cancer (GC).

As shown in Figure 4, the sensitivity analysis revealed that all included studies had no obvious influence on the pooled RR values of CDH17 expression in GC patients or the prognosis of GC. With the exception of the studies associated with TNM clinical stage, the contour-enhanced funnel plots were symmetric, thereby indicating no publication bias (TNM stage: P<0.05; Figure 5).

### Results of meta-regression analysis

**Figure 6:**
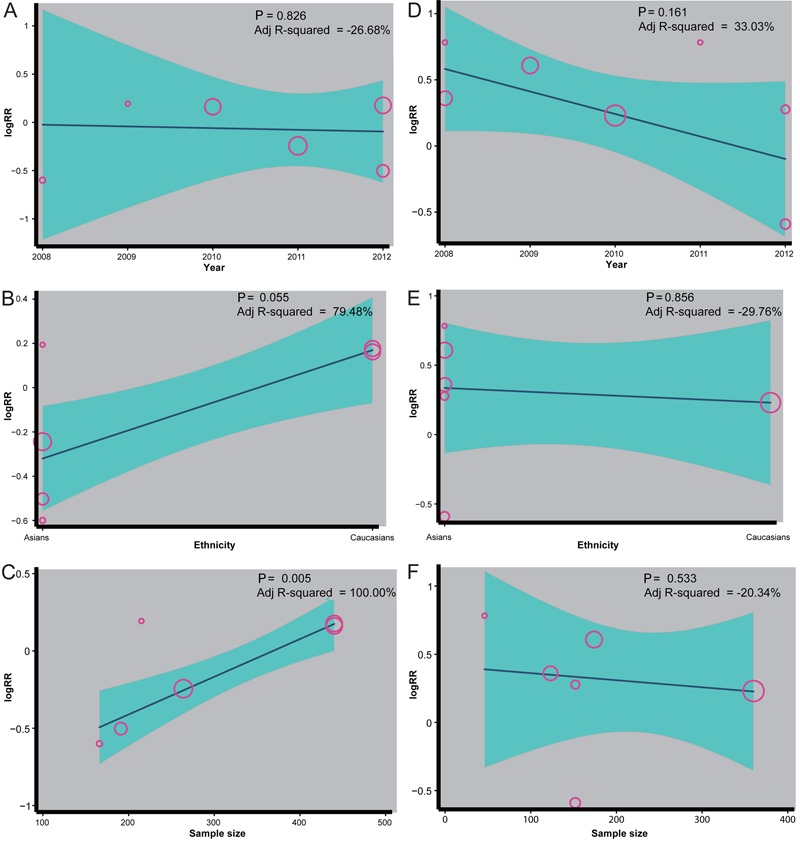
Meta-regression analyses of the Lauren classification (left panels) and the 5-year survival rate (right panels) of patients with gastric cancer (GC) based on the nine included studies.

The univariate meta-regression analysis of the Lauren classification and 5-year survival rate of the GC patients showed that publication year, ethnicity, and sample size were not the main sources of heterogeneity or influencing factors of pooled RR (P>0.05) (Figure 6). Moreover, a multivariate meta-regression analysis demonstrated that publication year, ethnicity, and sample size were not the sources of heterogeneity (Tables 2 and 3).

**Table:**
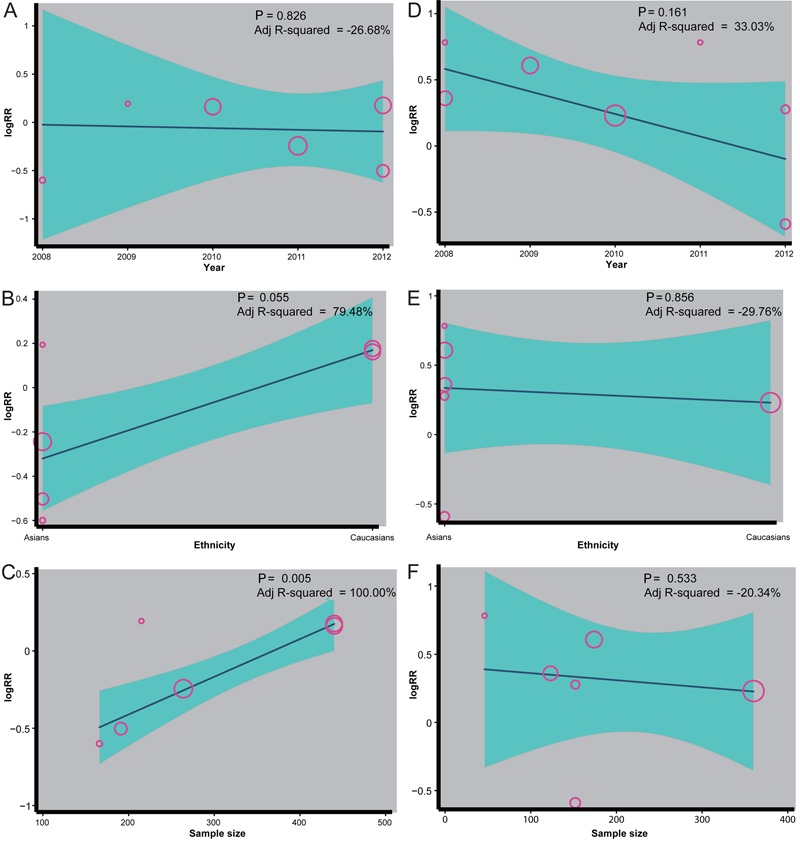

**Table:**
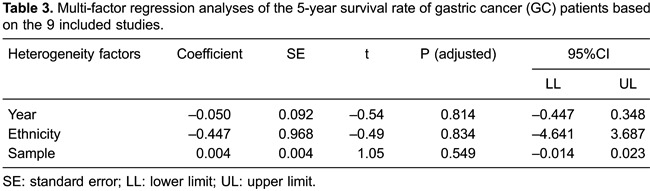

## Discussion

CDH17 is a unique member of the cadherin superfamily regulating intercellular adhesion. Several studies have reported that overexpression of CDH17 in GC is associated with a poorer prognosis, which is also associated with LNM and deeper invasion (11,16). However, there exists a discrepancy in that CDH17 has also been shown to be a prognostic marker for node-negative or early stage GC (15). In this regard, we conducted a meta-analysis to investigate the correlation of CDH17 with the clinicopathological features and prognosis of GC.

We discovered that CHD17 was a risk factor for the invasive depth of GC, revealing that CHD17 might participate in the invasion and metastasis of GC but can not be considered an independent predictor of GC prognosis. The molecular mechanisms underlying the regulation of GC growth by CDH17 are unknown. However, based on a recent report that indicated the existence of a trans-interaction between CDH17 and E-cadherin in enterocytes throughout intestinal epithelium development, we predict that CDH17 interacts with the Wnt pathway via coordination with E-cadherin or E-cadherin-related partners (29,30), although a previous study reported that targeting CDH17 inactivated the Wnt/β-catenin signaling pathway in hepatocellular carcinoma (31). Moreover, Wnt signaling-facilitated gastric carcinogenesis was previously reported in transgenic animal models (32).

The specific mechanisms by which CDH17 exerts a potential oncogenic role through the Wnt/β-catenin pathway in GC include a decrease in the phosphorylation of glycogen synthase kinase-3β and β-catenin, together with a simultaneous increase of retinoblastoma protein and decrease of cyclin D1, leading to an inhibition of cell proliferation (7). Additionally, the knockdown of CDH17 in GC cell lines (AGS and MKN-45) led to nuclear extravasation or cytoplasmic sequestration together with the potential degradation of β-catenin via the Wnt signaling pathway, which reduced the transactivation activity of lymphoid enhancer factor (LEF)/T-cell factor (TCF) transcription factors. This indirectly modulated cell proliferation and apoptosis (33).

Previous studies reported that intestinal metaplasia is a premalignant lesion (34-36) involving the cumulative loss of expression of differentiation or adhesion protein biomarkers such as CDH17, MUC13m, REG4, and LGALS4. This is thought to induce the disorganization of tissue architecture, which, together with cellular dedifferentiation, enhances carcinogenesis (11). These studies provide evidence for the hypothesis that CDH17 expression could be an important biomarker of gastric tissue malignancy. Contrary to previous studies reporting that the overexpression of CDH17 in GC could be regarded as an independent predictor for poor prognosis, we failed to find a correlation between CDH17 and GC prognosis. We also found that the positive expression of CDH17 in diffuse GC was significantly higher than in intestinal-type GC, suggesting that CDH17 might be considered a diagnostic criterion in distinguishing between the two categories of GC, though the reliability of this needs further investigation.

Some limitations potentially influenced the overall results of this meta-analysis. For instance, the small sample size for several outcomes indicated a trend for some results but could not be statistically significant. Additionally, data about age and gender were incomplete in some of the included studies, which could influence the overall findings.

In summary, we found that CDH17 might be associated with the early development of GC and that it is clearly involved in local invasion of GC tumors. However, we failed to find a role for CDH17 in GC progression to later stages and did not establish any links to overall survival in GC.
